# Social Needs Screening Tools for Clinical Populations in Australia and New Zealand: A Scoping Review and Critical Analysis

**DOI:** 10.1111/hex.70626

**Published:** 2026-02-26

**Authors:** Isabelle Weld‐Blundell, Yvonne C. Learmonth, Marlena Klaic, Jodi Haartsen, Darshini Ayton, Anne Kavanagh, Megan R. Hawkins, Claudia H. Marck

**Affiliations:** ^1^ Disability and Health Unit, The Melbourne School of Population and Global Health The University of Melbourne Melbourne Australia; ^2^ School of Health Sciences The University of New South Wales Sydney Australia; ^3^ School of Allied Health (Exercise Science) Murdoch University Perth Australia; ^4^ Perron Institute University of Western Australia, QEII Medical Centre Perth Australia; ^5^ Melbourne School of Health Sciences The University of Melbourne Melbourne Australia; ^6^ Alfred Health Monash University Melbourne Australia; ^7^ The School of Public Health and Preventive Medicine Monash University Melbourne Australia

**Keywords:** health equity, healthcare, social care, social determinants of health, social prescribing, social services

## Abstract

**Background:**

Social determinants of health account for approximately 50% of health outcomes, yet social needs are rarely assessed as part of routine clinical care. We aimed to conduct a scoping review of screening tools for assessing social needs within clinical practice in Australia and New Zealand.

**Methods:**

This scoping review was conducted according to our preregistered protocol (https://osf.io/d6evu). We searched scientific and grey literature for Australian or New Zealand studies that developed and/or evaluated social needs screening tools for adult patients. Extracted data included tool characteristics, validity, comprehensiveness, and proposed interventions. Actionability, that is, detailed specification of behaviour, was assessed using the Action, Actor, Context, Target and Time framework.

**Results:**

Eight studies were included, describing five Australian screening tools (none from New Zealand), with varied characteristics and validity measures. Tools frequently screened for employment, economic stability, housing, support systems, and social and community context. Two tools covered all social need domains, and one provided detailed behavioural specifications. However, none outlined referral pathways for identified needs.

**Conclusions:**

We identified five social needs screening tools. Most lacked comprehensiveness and actionability, and none integrated referral pathways. While these tools may represent a first step in identifying social needs in clinical care, addressing these gaps is essential for meaningful impact.

**Patient or Public Contribution:**

Our research team comprised people living with chronic health conditions, clinicians, researchers, and social epidemiologists who all contributed to study design, conduct and interpretation of data.

**Abbreviations**
AIHW: Australia Institute of Health and Welfare
BRFS: brief risk factor survey
FUST: Flinders University Social Health History Screening Tool
MS: multiple sclerosis
NEAT: nursing equity assessment tool
PRISMA‐Sc: preferred reporting items for systematic reviews and meta‐analyses extension for scoping reviews
SDOH: social determinants of health
SDoHST: social determinants of health screening tool
SIREN: social interventions research & evaluation network
STBH‐Q: steps to better health questionnaire

## 1 Introduction

Social determinants of health (SDOH) – the conditions in which people are born, live and work – play a critical role in shaping health outcomes [1]. These determinants are influenced by broader policy, economic, cultural and environmental factors. In Australia, evidence suggests that inequalities in SDOH are widening, disproportionately affecting those experiencing socioeconomic disadvantage and contributing to poorer health outcomes over time [2, 3, 4]. There is a critical need to consider SDOH if we are to address existing inequities and improve health outcomes for all [5]. At an individual level, unmet needs regarding social determinants of health, such as housing instability, food insecurity, lack of transport or social isolation, have been termed ***social needs*** [6]. Internationally, there is growing recognition that identifying and addressing social needs should be a core component of healthcare delivery [7, 8]. Despite accounting for at least 50% of health outcomes [1, 9, 10], social needs often receive less attention than clinical interventions in routine care [11, 12].

Screening for social needs in clinical settings has shown promise in improving health outcomes [13, 14, 15]. Tools like the HEEADSSS (Home, Education/Employment, Eating, Activities, Drugs, Sexuality, Suicide/Depression and Safety) assessment used in adolescent health [16], and the WE CARE (Well Child Care, Evaluation, Community Resources, Advocacy, Referral, Education) tool in paediatrics [17], demonstrate how structured screening can identify unmet needs and facilitate referrals to appropriate services. Furthermore, economic modelling from the United States indicates screening for social needs and judicious use of referral services is cost effective [18]. In Australia, it is recognised that integrated health and social care is crucial to meeting the needs of disadvantaged groups [19]. However, while addressing social needs through social prescribing (connecting the health, social and community sectors) has recently shown promise [20], Australian research is still limited [21]. The mechanisms through which social needs interventions in healthcare settings improve health and wellbeing may include: (1) reduced social needs; (2) reduced stress and anxiety; (3) improved quality of care and care effectiveness and (4) reduced care provider burnout [22]. However, comprehensive screening for social needs is not yet standard practice [23], leading to missed opportunities to address factors that significantly impact patient health and compound health inequities.

Most existing reviews focus on tools developed and tested in the United States [24, 25, 26, 27]. Given the substantial differences in healthcare systems, funding models, and social service infrastructure, the applicability of these tools to publicly funded systems like those in Australia and New Zealand is uncertain. It is well established that clear specification of behaviour (actionability) aids implementation in practice [28], however this is rarely assessed in reviews of social needs screening tools. When behaviours, such as the utilisation of a screening tool in clinical settings, are described in terms of what, who, when, where and how, they are more actionable and hence more likely to be implemented [29]. Furthermore, many reviews are limited to specific populations, such as paediatric [30] or cardiovascular patients [31], rather than offering a broad overview of tools suitable for general clinical use. Hence, it remains unclear whether comprehensive, valid, and actionable screening tools exist for assessing social needs across populations and in the Australian and New Zealand clinical contexts.

This scoping review aims to identify screening tools that assess a range of social needs in adult clinical populations in Australia and New Zealand. We assessed the validity (if evaluated), proposed interventions (if reported), comprehensiveness (number of social needs assessed) and actionability (specificity of behaviour) of each screening tool. A better understanding of the availability and characteristics of screening tools for social needs, will assist healthcare providers and services in their selection and implementation of appropriate tools to assess social needs.

## 2 Methods

The scoping review was conducted according to our preregistered protocol (Open Science Framework https://osf.io/d6evu) and reported in accordance with the Preferred Reporting Items for Systematic Reviews and Meta‐Analyses Extension for Scoping Reviews (PRISMA‐Sc). Our research team comprising people living with chronic health conditions, clinicians, researchers, and social epidemiologists informed the methodology. Specifically, frameworks, the search terms, selection criteria, data extraction items (e.g. administration time and setting), and assessments of tool content (actionability and comprehensiveness), were informed by the team members through meetings and iterative feedback rounds. Team members with lived experience of chronic illness further emphasised the importance of referral mechanisms in place once unmet social needs are identified.

### 2.1 Selection Criteria

We included Australian or New Zealand studies published from 2014, that focused on the development and/or evaluation of a screening tool intended to identify SDOH or social needs (non‐medical factors that could impact on health access or health outcomes) in an adult patient population in a clinical care context. The timeframe was chosen to capture recent advancements in the field, ensuring the synthesis reflects contemporary knowledge, making the findings more relevant and applicable to current and future research. As our focus was on the Australian and New Zealand context, we only included documents written in English language. For inclusion, screening tools had to assess three or more of the following social need domains adapted from the Henriksen et al. framework [24]: economic stability, education, social and community context, healthcare access, neighbourhood and physical environment, food, or health behaviours. Tools may also include other items related to medical factors. We excluded reviews, editorials, opinion pieces, commentaries, conference abstracts, dissertations, and studies with no publicly accessible screening tool. Studies were excluded if they were only intended to screen paediatric patient populations or non‐patient populations (e.g. veterans, students), or be administered for research purposes or population‐based programmes.

### 2.2 Information Sources and Search

In October 2024, we searched the scientific and grey literature. We searched 7 scientific databases MEDLINE (Ovid), Embase (Ovid), PsycINFO (Ovid), Health and psychosocial instruments (Ovid), CINAHL (EBSCOhost), Scopus and Web of Science [32]. To identify relevant grey literature, we searched Social Interventions Research & Evaluation Network (SIREN) and Trip Medical Database.

### 2.3 Search Strategy

The search terms and strategies were produced in consultation with a librarian (Appendix A). We ran pilot searches, confirmed that our search strategy was capturing known relevant studies, and made adjustments as needed. Furthermore, we cross‐checked our results against the references of relevant reviews (Appendix B) to ensure we did not miss any eligible studies.

### 2.4 Selection of Articles

Records identified from the search were recorded using reference management software Endnote 21, and imported into the review management platform Covidence, where duplicates were removed. Two researchers independently screened all titles and abstracts of identified records, and then independently assessed full‐text articles for inclusion according to the pre‐specified selection criteria. Any discrepancies were resolved by mutual agreement or a third researcher. Where relevant protocols were identified, we contacted authors for associated reports or (in‐press) publications.

### 2.5 Data Extraction

Two researchers independently extracted data items into an Excel template for more than half of the included studies (60%). The two researchers then discussed and resolved any discrepancies in extracted data items and scoring, before one researcher extracted data for the remaining studies with input from the second researcher as needed. The data extraction template included study characteristics: screening tool name, tool development method, whether each of the screening tools have been piloted and/or validated in any clinical population, number of items in tool, time to administer, target population, target user, intended setting and delivery, proposed interventions to address corresponding social needs identified by the tool, comprehensiveness, and actionability. Appendix C provides a list of extracted data items with definitions.

### 2.6 Assessment of Tool Content

Comprehensiveness was assessed as the number of social needs included in each screening tool. We considered frameworks from Dalgren [33], the Australia Institute of Health and Welfare (AIHW) [34], and Henrikson [24], and ultimately adopted the latter as the categorisation correlated well with SDOH at an individual level (i.e. social needs). The questions included in the screening tool were extracted, and categorised into social need domains and subdomains based on Henriksen et al.'s framework which was developed from literature, expert input, and multiple frameworks [35, 36]. Our team adapted Henrikson et al.'s framework to add a health behaviours domain, in line with several other frameworks for SDOH [33, 34], with the caveat that health behaviours are often consequences of SDOH. There were seven domains: economic stability, education, social and community context, healthcare access, neighbourhood and physical environment, food and health behaviours. Questions screening for physical and/or mental health were included in the count of number of questions but not considered social determinants and therefore not categorised into social domains.

The actionability of each screening tool was assessed using the Action, Actor, Context, Target, Time (AACTT) framework [28]. The AACTT framework is widely used in implementation science to specify or evaluate behavioural interventions in healthcare settings. The ‘action’ component was operationalised as the behaviour(s) involved in using the screening tool (e.g. providing the screening tool to patients for self‐completion). ‘Actor’ was operationalised as who was responsible for implementing the screening tool into practice (e.g. administrative staff), ‘context’ as where and in what circumstances the tool should be administered (e.g. waiting room), ‘target’ as who should be screened (e.g. patients new to service), and ‘time’ as when the screening tool should be administered (e.g. before first clinical assessment). For each of the tools, we reviewed the associated reports and supplementary material to assess if there were instructions about who should do what, in what context, to whom, and when. We based the AACTT assessment on the tool's intended design, which is not necessarily the same as its pilot study; that is, pilot study may have been limited to a specific patient demographic but the tool is intended for all patients, therefore the score for the Actor component was determined by how well ‘all patients’ were described. The AACTT items were each scored 0 = element not mentioned; 1 = element mentioned but nonspecific; or 2 = element mentioned, and specific detail provided (i.e., replicable). These five items produced a mean score between 0 (not specific) and 2 (highly specific). Higher scores are thought to better facilitate implementation of a healthcare intervention.

### 2.7 Data Synthesis

Study characteristics were presented in a table. Proposed interventions accompanying the screening tools were summarised descriptively. We assessed the number of social needs assessed in each screening tool (comprehensiveness), and displayed the results in a heatmap. We also synthesised the actionability of each tool based on the AACTT implementation framework, and displayed the AACTT scores in a heatmap.

## 3 Results

**Figure 1:**
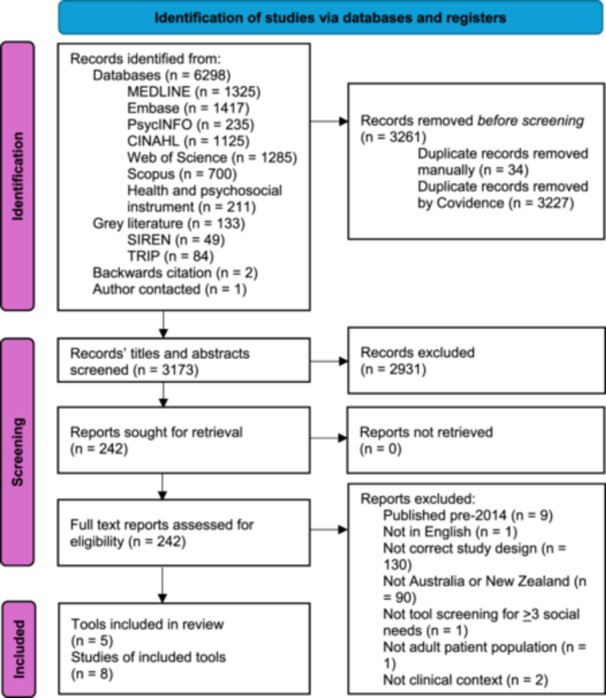
Preferred reporting items for systematic reviews and meta‐analyses flow diagram of included articles.

The searches yielded 3173 records after removal of duplicates, 2931 of which were then excluded after title and abstract screening. A further 234 studies were excluded after full‐text screening. Finally, eight articles describing five screening tools were included in this review [37, 38, 39, 40, 41, 42, 43, 44]. Browne‐Yung et al. used the Flinders University Social Health History Screening Tool (FUST) [37] and Neadley et al. used a modified version of FUST with simplified language [40]. The Nursing Equity Assessment Tool (NEAT) is described in two articles, one of which was sourced from the authors [38, 39]. The Brief Risk Factor Survey (BRFS) is also described in two articles [43, 44]. The Steps to Better Health Questionnaire (STBH‐Q) [41] and Social Determinants of Health Screening Tool (SDoHST) [42] were each described in one article. Figure 1 outlines the results of searching and screening in accordance with PRISMA‐Sc guidelines [45].

### 3.1 Characteristics of Included Tools

All included studies were Australian and were published in 2019 or later (Table 1). The number of questions per screening tool ranged from 12 to 25. Patients in general were the intended target for three of the tools: FUST, STBH‐Q, SDoHST. The NEAT was designed for people with cancer, and BRFS for people who are pregnant. Three of the screening tools – FUST, STBH‐Q, BRFS – were intended to be self‐completed by patients, whereas NEAT was intended to be used by nurses during consults. The average time to administer the screening tools was reported for two tools; 6 min for FUST and 3.9 min for NEAT. Three tools aimed to assess or measure individual level SDOH (FUST, STBH‐Q, SDoHST), while the NEAT described more broadly assessing 'medical and non‐medical factors known to increase a cancer patient's risk of complex care needs due to systemic inequities' [38] and the BRFS described assessing 'psychosocial and socio‐economic risk [43].'

**Table 1:** Characteristics of studies included in our scoping review of social needs screening tools.

First author, year, place	Tool	Tool development	Pilot testing	No. of items	Admin. time, mins	Target population	Target setting	Admin. method	Tool validation (methods used to validate tool)	SDOH variables assessed						
										Econ	Edu	SCC	HCC	NPE	Food	HB
Browne‐Yung (2019), Australia	Flinders University Social Health History Screening Tool (FUST)	Initial draft developed based on a standardised social history electronic form, theoretical frameworks and the research group's expertise. Refined through focus groups with health consumer advocates and interviews with clinicians.	50 new patients self‐completed the tool before first clinical consultation in anxiety disorder or sleep disorder clinics and later provided feedback	25	6	Patients	Clinical settings	Self‐complete; paper	**Qualitatively** validated for acceptability, face/content validity, and feasibility through focus groups, interviews, and questionnaires with health consumers, patients, and clinicians. Patients found questions easy to read and not overly sensitive. Clinicians provided positive feedback and recommended use especially for new patient consultations.	X	X	X	X	X	X	X
Neadley (2021), Australia	Modified version of FUST	Minor modifications were made to FUST to simplify the language of some questions	37 patients with diabetes mellitus, chronic obstructive pulmonary disease or heart failure who had been recently discharged from hospital self‐completed the tool	25	10	Patients	Clinical settings	Self‐complete; paper	Not validated	X	X	X	X	X	X	X
Chung (2023, 2024), Australia	Nursing Equity Assessment Tool (NEAT)	Draft checklist developed based on literature review and qualitative focus groups with 100 nurses. Refined through iterative feedback rounds from said focus groups and from pilot testing with 30 nurses on hypothetical scenarios.	30 nurses tested the checklist on 3 hypothetical case studies, and 5 clinical nurse consultants tested the checklist in consultations with 50 newly diagnosed cancer patients at a specialised cancer hospital	15	3.9	Newly diagnosed patients with cancer	Oncology setting	Specialist cancer nurses; verbal, stored in the electronic medical record	**Quantitatively validated** for predictive capacity. NEAT showed good agreement with evidence‐based patient reported outcome measures.	X	X	X	X	X		X
									**Qualitatively validated**: Preliminary content and face validity demonstrated through focus groups with 100 nurses and case study testing where 30 nurses tested the tool on 3 hypothetical case studies until no further modification was necessary. nurses pilot testing the tool on 50 newly diagnosed cancer patients confirmed content and face validity, and provided preliminary utility data. Clinical utility (appropriateness, acceptability, and practicability) was confirmed through semi‐structured interviews with 37 newly diagnosed patients and 7 clinical nurse consultants.							
Oster (2023), Australia	Steps to Better Health Questionnaire (STBH‐Q)	Draft questionnaire generated based on literature review and focus groups with potential end users (members of a medical society or community services network). Further refined through feedback from 22 participants on their understanding of each item.	330 adult members of the general community self‐completed STBH‐Q online	16	NR	NR	A range of settings, such as primary health care or via social prescribing programmes	Self‐complete; unclear	**Quantitatively validated.** Strong construct validity Good reliability as assessed via internal consistency. Modest to moderate strength correlations between the STBH‐Q and EQ‐5D‐5L quality of life measure. **Qualitatively validated** Theoretical validation through focus groups on clarity, relevance, and comprehensiveness of items leading to further modifications Semantic validation with 22 participants further improved the tool through minor wording changes to ensure the tool was practical, clear, and inclusive	X	X	X	X	X	X	X
Poirier (2023), Australia	Social Determinants of Health Screening Tool (SDoHST)	Draft tool developed based on literature review. Refined according to patient and stakeholder feedback.	5 adult patients accessing cancer services piloted SDoHST and discussed understanding, comprehension, and acceptability in a focus group. 9 stakeholders piloted SDoHST and discussed its content validity and scientific merit in a focus group.	12	NR	Adults with a chronic disease predominantly treated in hospital	Healthcare settings, particularly hospitals	NR	**Qualitatively validated** through piloting the instrument in a patient group and stakeholder group. Content analysis and cognitive debriefing of the focus group recommendations informed questionnaire modifications.	X		X		X	X	X
Price (2017, 2019), Australia	Brief risk factor survey (BRFS)	Draft survey developed based on antenatal and home visiting literature	166 pregnant women attending antenatal clinics at 2 metropolitan hospitals (Price 2017), and 735 pregnant women attending 10 public birthing hospitals (Price 2019) self‐completed BRFS in waiting room	16	NR	Pregnant women	Waiting rooms of antenatal clinics in public hospitals	Self‐complete	**Quantitatively validated**. BRFS count was associated with increased risk reported in antenatal interviews. High uptake and completion rates demonstrated feasibility and acceptability for collection by non‐clinical staff in antenatal settings.	X	X	X		X		X

#### 3.1.1 Tool Development and Validation

Almost all screening tools were developed using stakeholder involvement, however the extent of stakeholder involvement varied. The NEAT tool was developed through repeated, iterative feedback rounds with patients and stakeholders until no further modifications were required, while FUST, STBH‐Q and SDoHST were refined based on single rounds of stakeholder feedback. The BRFS was the only tool not informed by stakeholder feedback, but was refined based on assumptions drawn from response patterns and comparisons with clinical data [43, 44].

Both FUST and SDoHST were qualitatively validated. FUST was validated for acceptability, face and content validity, and feasibility through focus groups, interviews and questionnaires with patients and clinicians [37]. SDoHST was piloted in patient and stakeholder groups, and the content analysis and cognitive debriefing of the focus group feedback informed tool modifications [42].

BRFS was quantitatively validated, with the BRFS count associated with increased risk reported in antenatal interviews, demonstrating predictive validity [43]. High uptake and completion rates when administered by non‐clinical staff in antenatal settings demonstrated its feasibility and acceptability.

NEAT and STBH‐Q were both qualitatively and quantitatively validated. NEAT showed good agreement with evidence‐based measures, however predictive capacity should be interpreted with caution in the context of low statistical power [38]. In terms of qualitative validation, content and face validity was confirmed through nurses pilot testing NEAT with cancer patients, and clinical utility (appropriateness, acceptability and practicability) was then confirmed through interviews with patients and nurses [38]. STBH‐Q showed strong construct validity, good reliability, and modest‐to‐moderate correlations with quality of life measures [41]. Qualitative validation was demonstrated through focus groups on clarity, relevance, and comprehensiveness of items leading to tool modifications, and through participants making minor changes to wording.

#### 3.1.2 Pilot Studies

Most studies were piloted, either in simulated scenarios or real‐world environments. In Browne‐Yung (2019)'s pilot study of the FUST, none of the 50 patients indicated any social needs and therefore the tool has not been validated within a patient population with social needs [37]. In addition, while Oster et al. did not test the STBH‐Q in a clinical population (despite this being the target population), 44% of their participants had chronic health issues/disability [41]. Almost all pilot studies were conducted with a specific population in a specific context [37, 38, 39, 40, 42, 43, 44], and hence the tools may not be relevant to other patient populations.

#### 3.1.3 Social Needs Interventions

None of the included articles proposed specific interventions or referral pathways for identified needs, although most noted the importance of addressing identified needs and briefly mentioned ‘referral’ in their discussion. Chung et al. was the only study in which patients with identified needs were referred to services as part of the study, although the specifics of these referrals were not published [38]. In Browne‐Yung's study, participants in the pilot study noted that the results of the screening tool were not discussed in their consultation and patients were unsure whether the clinicians had reviewed their responses [37].

### 3.2 Comprehensiveness (as Measured by Domains)

**Figure 2:**
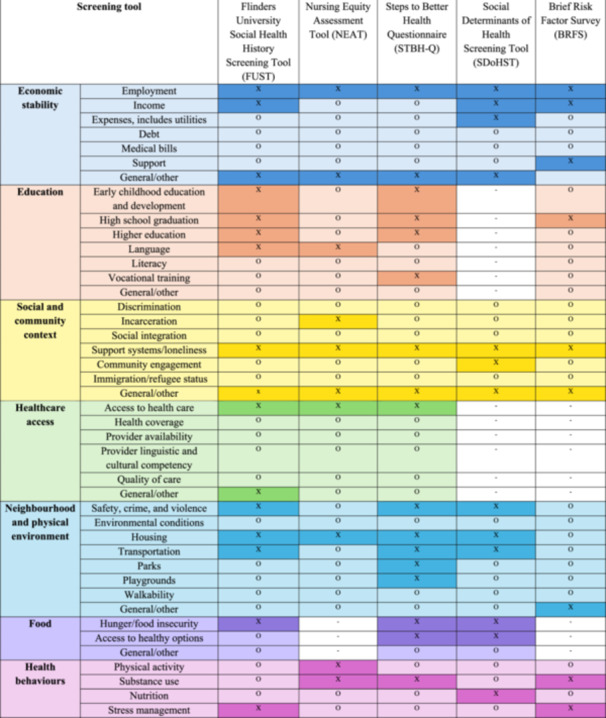
Heatmap of SDoH domains per screening tool (i.e. comprehensiveness). Dark colour (marked X): subdomain is addressed; light colour (marked O): domain but not subdomain is addressed; white (marked ‐): domain nor subdomain is addressed.

Screening tools included questions that covered between five and seven social needs domains, with FUST and STBH‐Q addressing all seven domains (Figure 2). All tools assessed at least one aspect of economic stability, social and community context, neighbourhood and physical environment, and health behaviours (*n* = 5, 100%), followed by education (*n* = 4, 80%). Examples of how economic stability were assessed include an item from the NEAT: 'Does the patient have financial concerns which will require referral? (yes/no),' and from the FUST: 'How would you say you are managing financially at the moment? (living comfortably/getting by/finding it difficult).' Healthcare access and food were assessed less frequently (*n* = 3, 60%). The safety, crime, and violence subdomain encompassed both neighbourhood violence and interpersonal violence. Three tools screened for safety, all of which specifically screened for domestic or family violence (FUST, STBH‐Q, SDoHST). Examples of how this was assessed include this item from FUST: 'How safe do you feel in your home/neighbourhood? (Very safe/Somewhat safe/Neither safe nor unsafe/Unsafe/Very unsafe)' and from the SDoHST: 'Did you or anyone in your family feel unsafe or afraid in your home? (yes/no).' The NEAT and BRFS did not consider safety, however, nurses recommended the addition of an item to address elder abuse and intimate partner violence to NEAT [38].

### 3.3 Actionability of Tools (as Measured by AACTT)

**Figure 3:**
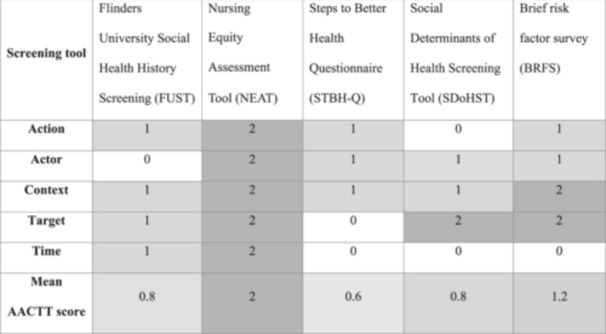
Heatmap of action, actor, context, target, time (ACCTT) framework scores (i.e. actionability) of each social needs screening tool.

Figure 3 shows the actionability scores of each tool, with additional details available in appendix D. The NEAT had the highest mean AACTT score of 2, followed by the Brief Risk Survey (mean 1.2), FUST and SDoHST (mean 0.8) and the STBH‐Q (mean 0.6). Timing of tool administration was only specified by the FUST ('before their first clinical consultation') and NEAT ('between first consultation and establishment of treatment plan') but instructions were unclear or missing on whether social needs should be assessed repeatedly and with what frequency. While context was often mentioned, the specifics of where or in what circumstance the screening tool should be implemented was lacking.

## 4 Discussion

This scoping review is the first to provide an overview of screening tools for a range of social needs for use in clinical settings in Australia and New Zealand, where many healthcare services are supported through a public healthcare system. Five multi‐domain screening tools were identified – far fewer than the over 20 tools identified in US‐based reviews [24, 25] – and these had variable methods of validation, comprehensiveness, and actionability. None of the articles specified referral pathways or interventions associated with the screening tool, although one reported that referrals would take place. Screening for social needs without taking appropriate action to respond to identified unmet needs may be more harmful than not screening at all [46, 47]. As such, there is currently no single screening tool that can be recommended for implementation across clinical settings in Australia and New Zealand.

### 4.1 Comprehensiveness

Since unmet social needs can have a compounding effect, and this can lead to an accumulating adverse impact on health, it is important to capture the range of social needs present in an individual's life [48]. The FUST and STBH‐Q were the most comprehensive tools in terms of the range of social needs domains assessed. However, this conclusion should be considered with the caveat that it is based on our categorisation of social needs using an adapted framework from Henrikson et al. It is essential to consider the potential for other categorisations of social needs. For example, water and sanitation are considered in Dalgren's framework [33], and health literacy and beliefs are considered their own category in the AIHW framework [34]. Hence, potential users should consider the most relevant domains and subdomains for their patient population when selecting an appropriate screening tool.

The most commonly assessed domains among the identified tools – economic stability, neighbourhood and physical environment, support systems, and general social and community context – differed slightly from the findings of US‐based reviews, which found physical environment [24, 25, 26], food insecurity [25, 26], and safety/violence [25] as the most frequently covered areas. In our review, only three tools screened for personal safety. While the NEAT did not include safety, the addition of an item to address elder abuse and intimate partner violence was recommended by nurses during evaluations [38]. Thus, the next iteration of NEAT may indeed screen for safety. This domain is pertinent to the Australian and New Zealand context, where one in four (23%) women in Australasia will experience intimate partner violence in their lifetime [49], and one in six (15%) elderly Australians will experience abuse in the community [50].

### 4.2 Actionability

Clear specification of behaviour (actionability) aids implementation of tools in clinical practice [28]. Four of the five screening tools lacked sufficient detail about actionability for effective implementation. The Action and Actor were often not specified, which could hinder initial implementation. In terms of the Target, most of the screening tools included in this review were intended for screening among patients in general. However, the NEAT was intended for patients with cancer, and BRFS for pregnant women. This is similar to previous reviews, with some tools found for pregnant patients [25] and patients with chronic disease such as cardiovascular disease [31]. The lack of detail regarding the intended Timing and Context of administration meant that users were not guided on when, how often, or where they should use the screening tool. For instance, authors would commonly state that screening tools are intended for patients in hospital clinics but not specify whether patients should complete these in the waiting room or at home prior to their appointment; details which can greatly influence the implementation of these tools as well as patients' answers. Such lack of detail may result in tools being used infrequently or at inappropriate times. Interestingly, although NEAT was not integrated into the electronic medical record in Chung et al.'s study, the authors mentioned that it would ideally be in the future [38].

### 4.3 Social Needs Interventions

Research highlights the importance of having community resources available to address identified social needs when conducting social needs screening [51]. However, the included screening tools lacked associated interventions and referral pathways to address identified needs, resulting in a lack of guidance on the next steps after screening. The pilot study by Browne‐Yung et al. demonstrates how screening tools without appropriate follow‐up may be ineffective and reduce trust in the healthcare service [37]. Participants reported that the results of the screening tool were not discussed in consultations, and patients were unsure if clinicians had reviewed their responses [37]. This absence of proposed interventions is notable when compared to a US review in which 18 of the 30 included articles proposed specific interventions to address social needs [25]. Assessing social needs without taking any action undermines the purpose of screening and can be more harmful to the patient, clinician, and therapeutic relationship than not screening at all [46, 47]. Hence, it is crucial that screening tools are implemented in conjunction with pathways and capabilities that address identified needs. If identified needs cannot be met, efforts should focus on building resources, services and capabilities to meet these needs, and actions taken to start this process should be communicated to the patient.

The research teams associated with two of the identified screening tools have produced work to further address social needs. In affiliation with the Victoria Department of Health, Chung et al.'s team has created an online platform named ‘WeCan’ (https://wecan.org.au/) to link people living with cancer to social services [52]. In Chung et al.'s study, patients with identified needs were referred to services, but the specifics of these referrals were not published [38]. In addition, Oster et al. produced an article titled ‘The process of co‐designing a model of social prescribing: An Australian case study' in 2024 [53]. While this article did not focus on the development or evaluation of the STBH‐Q, and therefore could not be included in our review, it used the STBH‐Q to perform a community needs assessment as part of a larger co‐design social prescribing programme in regional Australia [20]. Hence, both teams have produced additional work on how to address social needs, although these efforts were not covered in the articles included in this scoping review. Ideally, screening tools should be linked to such resources prior to implementation, however outlining social interventions in published literature may be challenging due to the local and time‐limited nature of many social services and programmes.

While some of the tools we identified focused on a specific population, there are benefits to broad‑population social needs screening tools. For example, these are more scalable and easier to integrate into routine care and electronic health records than multiple population‐specific instruments, reducing training requirements and importantly, providing standardised data for monitoring. Broad screening tools implemented systematically also normalises screening and potentially lowers stigma. It is important to recognise that addressing social needs is beyond the capacity of health services and clinicians alone; a broader integrative cross‐sectional approach is required. Social inequities are complex and commonly intersect, driven by the political landscape, and are ideally eased at a macro level through improving policies, economic, cultural and environmental conditions [5, 54]. However, since this is falling short in Australia, with inequities growing [2], a multilevel approach including healthcare providers may be a strategy some healthcare providers want to adopt to improve health outcomes for their patients [55].

### 4.4 Strengths and Limitations

This is the first study to conduct a thorough review and comparison of available screening tools to address social needs in Australia and New Zealand. In addition, registering our protocol on Open Science Framework enhances transparency, and developing our search strategy in consultation with a librarian improves methodological rigour. The methodological rigour and interpretation of findings were further strengthened by input from our research team comprising people living with chronic health conditions, clinicians, researchers, and social epidemiologists. While our assessments regarding comprehensiveness and actionability are not based on universal standards, these assessments were based on well‐known frameworks in the field that are widely used for this purpose. It is important to consider the findings of this review within the context of its scope, as our focus was on Australian and New Zealand articles (although we did not find any from New Zealand). Hence, our findings may be of less relevance to healthcare contexts in countries other than Australia.

### 4.5 Implications and Future Directions

It is well‐established that social needs impart significant influence on health outcomes. Several reviews have demonstrated that addressing social needs (e.g., housing instability, food insecurity) through referrals to appropriate services often eases the identified social need, can improve health outcomes such as lowering blood pressure and cholesterol levels, and reduce healthcare utilisation and costs [13, 14, 15]. The present review has identified what screening tools already exist for assessing social needs among patient populations in Australia and New Zealand, which is the first step to addressing social needs in practice. To improve the evidence‐base and benefit the broader healthcare community, we encourage clinicians to publish and validate any 'in‐house' tools which may until now be unpublished or unvalidated. Further improvements, including to the comprehensiveness and actionability of tools are needed. In addition, providing referral pathways and interventions, as well as assessing the potential costs and benefits of assessing and addressing social needs in specific populations would be a necessary step before implementing these tools. Ultimately, creating integrated models of care where social needs are addressed in meaningful person‐centred ways will be complex but imperative to improve health care and outcomes. Social needs should be acknowledged, with clinicians intervening where possible as exemplified in Vanjani et al.'s article [54] or referring to services and programmes. When services are lacking, there should be pathways to advocate for further resource allocation.

## 5 Conclusion

Social needs are recognised, important drivers of health outcomes and inequities. Assessment or screening of unmet social needs in clinical settings offers a practical route to identify patients who may benefit from additional support or referrals, such as to social services. This review identified five multi‐domain screening tools for assessing social needs in Australian clinical settings, no tools were identified from New Zealand. Existing instruments are limited by variable comprehensiveness, inconsistent actionability and a lack of clearly defined referral pathways and intervention links. To ensure screening translates into meaningful improvements in health and equity, future work should: validate and refine tools across diverse clinical and community contexts; co‑design instruments and referral processes with service users; embed tools within clinical workflows and electronic systems; and evaluate the downstream effects of screening on service access, health outcomes and cost‑effectiveness.

## Author Contributions

**Isabelle Weld‐Blundell:** data curation, formal analysis, funding acquisition, investigation, methodology, validation, visualization, writing – original draft. **Yvonne C. Learmonth:** data curation, formal analysis, funding acquisition, methodology, writing – review and editing. **Marlena Klaic:** funding acquisition, methodology, writing – review and editing. **Jodi Haartsen:** funding acquisition, methodology, writing – review and editing. **Darshini Ayton:** funding acquisition, methodology, writing – review and editing. **Anne Kavanagh:** funding acquisition, methodology, writing – review and editing. **Megan R. Hawkins:** methodology, writing – review and editing. **Claudia H. Marck:** conceptualiztion, formal analysis, funding acquisition, methodology, project administration, supervision, validation, writing – original draft, writing – review and editing.

## Ethics Statement

The authors have nothing to report.

## Consent

The authors have nothing to report.

## Conflicts of Interest

The authors declare no conflicts of interest.

## Supporting information

Appendix_A.

Appendix_B.

Appendix_C.

Appendix_D.

## Data Availability

Data sharing not applicable to this article as no datasets were generated or analysed during the current study.
