# Supplementary material for: Social Needs Screening Tools for Clinical Populations in Australia and New Zealand: A Scoping Review and Critical Analysis
Source: Health Expect. 2026 Feb 26;29(2):e70626. doi: 10.1111/hex.70626 (PMC12936985; doi:10.1111/hex.70626)
Supplement: Supplementary file 1 — Appendix_A. [file HEX-29-e70626-s001.docx]

Appendix A. Search strategies

Scientific databases searched 25/10/2024

| **Medline (Ovid)**    **1.** *"social determinants of health"/    **2.** ("social determinant*" or "social need*" or "social risk*" or "determinant* of health" or SDOH).ti,kf.    **3.** *socioeconomic factors/ or *economic stability/ or *low socioeconomic status/ or *poverty/ or *unemployment/ or (economic or socioeconomic or poverty or employment or unemployment or financial or "low income").ti,kf.    **4.** *education/ or (education or "health literacy").ti,kf.    ***5.* ***social isolation/ or *social vulnerability/ or *loneliness/ or *social environment/ or *social discrimination/ or *community participation/ or *community support/ or *social support/ or ("social support*" or "support network*" or "social exclusion" or "social network*" or "social isolation" or loneliness or discrimination or racism or incarcerat* or immigra* or refugee*).ti,kf.    **6. ***health services accessibility/ or ((health or healthcare) adj2 access*).ti,kf.    **7.** *residence characteristics/ or *home environment/ or *housing/ or *housing instability/ or *ill-housed persons/ or *violence/ or *domestic violence/ or *intimate partner violence/ or (neighborhood or neighbourhood or environment* or housing or transport* or violence).ti,kf.    **8.** *food insecurity/ or *food supply/ or ("food security" or "food insecurity").ti,kf.    **9. ***health behavior/ or ("health* behavior*" or "health* behaviour*" or lifestyle or nutrition or malnutrition or diet* or smoking or "physical activity" or exercise or sedentary).ti,kf.    **10.** (3 and 4 and 5) or (3 and 4 and 6) or (3 and 4 and 7) or (3 and 4 and 8) or (3 and 4 and 9) or (3 and 5 and 6) or (3 and 5 and 7) or (3 and 5 and 8) or (3 and 5 and 9) or (3 and 6 and 7) or (3 and 6 and 8) or (3 and 6 and 9) or (3 and 7 and 8) or (3 and 7 and 9) or (3 and 8 and 9) or (4 and 5 and 6) or (4 and 5 and 7) or (4 and 5 and 8) or (4 and 5 and 9) or (4 and 6 and 7) or (4 and 6 and 8) or (4 and 6 and 9) or (4 and 7 and 8) or (4 and 7 and 9) or (4 and 8 and 9) or (5 and 6 and 7) or (5 and 6 and 8) or (5 and 6 and 9) or (5 and 7 and 8) or (5 and 7 and 9) or (5 and 8 and 9) or (6 and 7 and 8) or (6 and 7 and 9) or (6 and 8 and 9) or (7 and 8 and 9)    **11.**  1 or 2 or 10    **12.** *needs assessment/ or *"surveys and questionnaires"/ or *mass screening/ or *risk assessment/    **13.** (screen* or "tool" or " checklist" or "needs assessment" or "questionnaire" or ((assess* or identif* or captur* or develop* or evaluat* or collect*) adj3 ("social determinant*" or "social need*" or "social risk*" or "determinant* of health" or SDOH))).ti,kf,hw.    **14.** 12 or 13    **15.**  11 and 14    **16.** limit 15 to yr="2014 -Current" |
| --- |
| **Embase (Ovid)**    **1.** *"social determinants of health"/ or *social needs/    **2.** ("social determinant*" or "social need*" or "social risk*" or "determinant* of health" or SDOH).ti,kf.    **3.** *socioeconomics/ or *economic stability/ or *economic status/ or *low socioeconomic status/ or *poverty/ or *income group/ or *lowest income group/ or *socioeconomic vulnerability/ or *employment status/ or *unemployment/ or *employment/ or *financial stress/ or (economic or socioeconomic or poverty or employment or unemployment or financial or "low income").ti,kf.    **4.** *education/ or *health literacy/ or (education or "health literacy").ti,kf.    ***5.* ***social isolation/ or *social vulnerability/ or *loneliness/ or *social environment/ or *social discrimination/ or *community participation/ or *community support/ or *social support/ or ("social support*" or "support network*" or "social exclusion" or "social network*" or "social isolation" or loneliness or discrimination or racism or incarcerat* or immigra* or refugee*).ti,kf.    **6. ***health care access/ or ((health or healthcare) adj2 access*).ti,kf.    **7.** *residence characteristics/ or *home environment/ or *housing/ or *housing instability/ or *homeless person/ or *violence/ or *domestic violence/ or *partner violence/ or *family violence/ or *exposure to violence/ or (neighborhood or neighbourhood or environment* or housing or transport* or violence).ti,kf.    **8.** *food insecurity/ or ("food security" or "food insecurity").ti,kf.    **9. ***health behavior/ or *****lifestyle/ or *****healthy lifestyle/ or ("health* behavior*" or "health* behaviour*" or lifestyle or nutrition or malnutrition or diet* or smoking or "physical activity" or exercise or sedentary).ti,kf.    **10.** (3 and 4 and 5) or (3 and 4 and 6) or (3 and 4 and 7) or (3 and 4 and 8) or (3 and 4 and 9) or (3 and 5 and 6) or (3 and 5 and 7) or (3 and 5 and 8) or (3 and 5 and 9) or (3 and 6 and 7) or (3 and 6 and 8) or (3 and 6 and 9) or (3 and 7 and 8) or (3 and 7 and 9) or (3 and 8 and 9) or (4 and 5 and 6) or (4 and 5 and 7) or (4 and 5 and 8) or (4 and 5 and 9) or (4 and 6 and 7) or (4 and 6 and 8) or (4 and 6 and 9) or (4 and 7 and 8) or (4 and 7 and 9) or (4 and 8 and 9) or (5 and 6 and 7) or (5 and 6 and 8) or (5 and 6 and 9) or (5 and 7 and 8) or (5 and 7 and 9) or (5 and 8 and 9) or (6 and 7 and 8) or (6 and 7 and 9) or (6 and 8 and 9) or (7 and 8 and 9)    **11.**  1 or 2 or 10    **12.** *needs assessment/ or *questionnaire/ or *mass screening/ or *risk assessment/    **13.** (screen* or "tool" or " checklist" or "needs assessment" or "questionnaire" or ((assess* or identif* or captur* or develop* or evaluat* or collect*) adj3 ("social determinant*" or "social need*" or "social risk*" or "determinant* of health" or SDOH))).ti,kf.    **14.** 12 or 13    **15.**  11 and 14    **16.** limit 15 to yr="2014 -Current" |
| **PsycINFO (Ovid)**    **1.** *"social determinants of health"/    **2.** ("social determinant*" or "social need*" or "social risk*" or "determinant* of health" or SDOH).ti,id.    **3.** *socioeconomic factors/ or *economic security/ or *socioeconomic status/ or *lower income level/ or *poverty/ or *employment status/ or *unemployment/ or (economic or socioeconomic or poverty or employment or unemployment or financial or "low income").ti,id.    **4.** *education/ or *health literacy/ or (education or "health literacy").ti,id.    ***5.* ***social isolation/ or *loneliness/ or *social environments/ or *social discrimination/ or *community involvement/ or *social support/ or ("social support*" or "support network*" or "social exclusion" or "social network*" or "social isolation" or loneliness or discrimination or racism or incarcerat* or immigra* or refugee*).ti,id.    **6. ***health care access/ or ((health or healthcare) adj2 access*).ti,id.    **7. ***neighborhoods/ or *home environment/ or *housing/ or *homeless/ or *violence/ or *domestic violence/ or *intimate partner violence/ or (neighborhood or neighbourhood or environment* or housing or transport* or violence).ti,id.    **8.** *food insecurity/ or ("food security" or "food insecurity").ti,id.    **9. ***health behavior/ or *****lifestyle/ or ("health* behavior*" or "health* behaviour*" or lifestyle or nutrition or malnutrition or diet* or smoking or "physical activity" or exercise or sedentary).ti,id.    **10.** (3 and 4 and 5) or (3 and 4 and 6) or (3 and 4 and 7) or (3 and 4 and 8) or (3 and 4 and 9) or (3 and 5 and 6) or (3 and 5 and 7) or (3 and 5 and 8) or (3 and 5 and 9) or (3 and 6 and 7) or (3 and 6 and 8) or (3 and 6 and 9) or (3 and 7 and 8) or (3 and 7 and 9) or (3 and 8 and 9) or (4 and 5 and 6) or (4 and 5 and 7) or (4 and 5 and 8) or (4 and 5 and 9) or (4 and 6 and 7) or (4 and 6 and 8) or (4 and 6 and 9) or (4 and 7 and 8) or (4 and 7 and 9) or (4 and 8 and 9) or (5 and 6 and 7) or (5 and 6 and 8) or (5 and 6 and 9) or (5 and 7 and 8) or (5 and 7 and 9) or (5 and 8 and 9) or (6 and 7 and 8) or (6 and 7 and 9) or (6 and 8 and 9) or (7 and 8 and 9)    **11.**  1 or 2 or 10    **12**. *screening/ or *screening tests/ or *needs assessment/ or *questionnaires/ or *risk assessment/    **13.** (screen* or "tool" or " checklist" or "needs assessment" or "questionnaire" or ((assess* or identif* or captur* or develop* or evaluat* or collect*) adj3 ("social determinant*" or "social need*" or "social risk*" or "determinant* of health" or SDOH))).ti,id.    **14.** 12 or 13    **15.**  11 and 14    **16.** limit 15 to yr="2014 -Current" |
| **CINAHL (EBSCO)**    (MM "Social Determinants of Health") or (MM "Social Problems")    TI ("social determinant*" or "social need*" or "social risk*" or "determinant* of health" or SDOH)    **3.** (MM "Socioeconomic Factors") or (MM "Economic Factors") or (MM "Low Socioeconomic Status") or (MM "Economic Status") or (MM "Poverty") or (MM "Employment Status") or (MM "Employment") or (MM "Unemployment") or (MM "Income") or (MM "Financial Stress") or TI (economic or socioeconomic or poverty or employment or unemployment or financial or "low income")    **4.** (MM "Education") or (MM "Health Literacy") or TI (education or "health literacy")    ***5.*** (MM "Social Isolation") or (MM "Social Deprivation") OR (MM "Social Environment") or (MM "Loneliness") or (MM "Discrimination") or (MM "Social Participation") OR (MM "Community Networks") or (MM "Community Support") or (MM "Support, Social") or (MM "Social Networks") or TI ("social support*" or "support network*" or "social exclusion" or "social network*" or "social isolation" or loneliness or discrimination or racism or incarcerat* or immigra* or refugee*)    **6.** (MM "Health Services Accessibility") or TI ((health or healthcare) N1 access*)    **7. (**MM "Residence Characteristics")  or (MM "Neighborhood Characteristics") or (MM "Housing") OR (MM "Housing Instability") or (MM "Homeless Persons") OR (MM "Homelessness") or (MM "Violence") OR (MM "Domestic Violence") OR (MM "Intimate Partner Violence") or TI (neighborhood or neighbourhood or environment* or housing or transport* or violence)    **8.** (MM "Food Security") OR (MM "Access to Healthy Foods") or TI ("food security" or "food insecurity")    **9.** (MM "Health Behavior") or (MM "Life Style") or TI ("health* behavior*" or "health* behaviour*" or lifestyle or nutrition or malnutrition or diet* or smoking or "physical activity" or exercise or sedentary)    **10.** (S3 and S4 and S5) or (S3 and S4 and S6) or (S3 and S4 and S7) or (S3 and S4 and S8) or (S3 and S4 and S9) or (S3 and S5 and S6) or (S3 and S5 and S7) or (S3 and S5 and S8) or (S3 and S5 and S9) or (S3 and S6 and S7) or (S3 and S6 and S8) or (S3 and S6 and S9) or (S3 and S7 and S8) or (S3 and S7 and S9) or (S3 and S8 and S9) or (S4 and S5 and S6) or (S4 and S5 and S7) or (S4 and S5 and S8) or (S4 and S5 and S9) or (S4 and S6 and S7) or (S4 and S6 and S8) or (S4 and S6 and S9) or (S4 and S7 and S8) or (S4 and S7 and S9) or (S4 and S8 and S9) or (S5 and S6 and S7) or (S5 and S6 and S8) or (S5 and S6 and S9) or (S5 and S7 and S8) or (S5 and S7 and S9) or (S5 and S8 and S9) or (S6 and S7 and S8) or (S6 and S7 and S9) or (S6 and S8 and S9) or (S7 and S8 and S9)    **11. S1 or S2 or S10**    12. (MM "Needs Assessment") or (MM "Risk Assessment") or (MM "Questionnaires")    13. TI (screen* or "tool" or " checklist" or "needs assessment" or "questionnaire" or ((assess* or identif* or captur* or develop* or evaluat* or collect*) N2 ("social determinant*" or "social need*" or "social risk*" or " determinant* of health" or SDOH)))    **14. S11 or S12**    14. **10 and** 13    **Limiters** - Publication Date: 20140101- |
| **Web of Science (Web of Knowledge)**    TI = ("social determinant*" or "social need*" or "social risk*" or "determinant* of health" or SDOH)    AK = ("social determinant*" or "social need*" or "social risk*" or "determinant* of health" or SDOH)    **3.** TI = (economic or socioeconomic or poverty or employment or unemployment or financial or "low income")    **4.** AK = (economic or socioeconomic or poverty or employment or unemployment or financial or "low income")    **5. 3 or 4**    **6.** TI = (education or "health literacy")    7. AK = (education or "health literacy")    **8. 6 or 7**    9. TI = ("social support*" or "support network*" or "social exclusion" or "social network*" or "social isolation" or loneliness or discrimination or racism or incarcerat* or immigra* or refugee*)    **10.** AK = ("social support*" or "support network*" or "social exclusion" or "social network*" or "social isolation" or loneliness or discrimination or racism or incarcerat* or immigra* or refugee*)    **11. 9 or 10**    **12.  TI =** ((health or healthcare) NEAR/1 access*)    **13. AK =** ((health or healthcare) NEAR/1 access*)    **14. 12 or 13**    **15.  TI =** (neighborhood or neighbourhood or environment* or housing or transport* or violence)    16.  **AK =** (neighborhood or neighbourhood or environment* or housing or transport* or violence)    **17. 15 or 16**    **18.** TI = ("food security" or "food insecurity")    19. AK = ("food security" or "food insecurity")    **20. 18 or 19**    **21. TI =** ("health* behavior*" or "health* behaviour*" or lifestyle or nutrition or malnutrition or diet* or smoking or "physical activity" or exercise or sedentary)    22. **AK =** ("health* behavior*" or "health* behaviour*" or lifestyle or nutrition or malnutrition or diet* or smoking or "physical activity" or exercise or sedentary)    23. 21 or 22    **24.** (#5 AND #8 AND #11) OR (#5 AND #8 AND #14) OR (#5 AND #8 AND #17) OR (#5 AND #8 AND #20) OR (#5 AND #8 AND #23) OR (#5 AND #11 AND #14) OR (#5 AND #11 AND #17) OR (#5 AND #11 AND #20) OR (#5 AND #11 AND #23) OR (#5 AND #14 AND #17) OR (#5 AND #14 AND #20) OR (#5 AND #14 AND #23) OR (#5 AND #17 AND #20) OR (#5 AND #17 AND #23) OR (#5 AND #20 AND #23) OR (#8 AND #11 AND #14) OR (#8 AND #11 AND #17) OR (#8 AND #11 AND #20) OR (#8 AND #11 AND #23) OR (#8 AND #14 AND #17) OR (#8 AND #14 AND #20) OR (#8 AND #14 AND #23) OR (#8 AND #17 AND #20) OR (#8 AND #17 AND #23) OR (#8 AND #20 AND #23) OR (#11 AND #14 AND #17) OR (#11 AND #14 AND #20) OR (#11 AND #14 AND #23) OR (#11 AND #17 AND #20) OR (#11 AND #17 AND #23) OR (#11 AND #20 AND #23) OR (#14 AND #17 AND #20) OR (#14 AND #17 AND #23) OR (#14 AND #20 AND #23) OR (#17 AND #20 AND #23)    **25.**  #1 OR #2 OR #24    26. TI = (screen* or "tool" or " checklist" or "needs assessment" or "questionnaire" or ((assess* or identif* or captur* or develop* or evaluat* or collect*) NEAR/2 ("social determinant*" or "social need*" or "social risk*" or "determinant* of health" or SDOH)))    AK = (screen* or "tool" or " checklist" or "needs assessment" or "questionnaire" or ((assess* or identif* or captur* or develop* or evaluat* or collect*) NEAR/2 ("social determinant*" or "social need*" or "social risk*" or "determinant* of health" or SDOH)))    **28. 26 or 27**    **29. 25 and 28**  **Refined By:Publication Years: 2024 or 2023 or 2022 or 2021 or 2020 or 2019 or 2018 or 2017 or 2016 or 2015 or 2014** |
| **Scopus (Elsevier)**    **1.** ( TITLE ( "social determinant*" OR "social need*" OR "social risk*" OR "determinant* of health" OR SDOH ) )    **2**. TITLE ( ( screen* or "tool" or " checklist" or "needs assessment" or "questionnaire" OR ( (assess* or identif* or captur* or develop* or evaluat* or collect* ) W/2 ( "social determinant*" or "social need*" or "social risk*" or "determinant* of health" or SDOH) ) ) )    3. 1 and 2    Limit 2014-2024    == 700    **3**. TITLE ( economic OR socioeconomic OR poverty OR employment OR unemployment OR financial OR "low income" )    **4**. TITLE ( education OR "health literacy" )    **5**. TITLE ( "social support*" or "support network*" or "social exclusion" or "social network*" or "social isolation" or loneliness or discrimination or racism or incarcerat* or immigra* or refugee* )    6. TITLE ( ( health OR healthcare ) W/1 access* )    7. TITLE ( neighborhood OR neighbourhood OR environment* OR housing OR transport* OR violence )    8. TITLE ( "food security" OR "food insecurity" )    9. TITLE ( "health* behavior*" or "health* behaviour*" or lifestyle or nutrition or malnutrition or diet* or smoking or "physical activity" or exercise or sedentary )    10. (3 and 4 and 5) or (3 and 4 and 6) or (3 and 4 and 7) or (3 and 4 and 8) or (3 and 4 and 9) or (3 and 5 and 6) or (3 and 5 and 7) or (3 and 5 and 8) or (3 and 5 and 9) or (3 and 6 and 7) or (3 and 6 and 8) or (3 and 6 and 9) or (3 and 7 and 8) or (3 and 7 and 9) or (3 and 8 and 9) or (4 and 5 and 6) or (4 and 5 and 7) or (4 and 5 and 8) or (4 and 5 and 9) or (4 and 6 and 7) or (4 and 6 and 8) or (4 and 6 and 9) or (4 and 7 and 8) or (4 and 7 and 9) or (4 and 8 and 9) or (5 and 6 and 7) or (5 and 6 and 8) or (5 and 6 and 9) or (5 and 7 and 8) or (5 and 7 and 9) or (5 and 8 and 9) or (6 and 7 and 8) or (6 and 7 and 9) or (6 and 8 and 9) or (7 and 8 and 9)    **11**. 1 or 10    **12**. 2 and 11 |
| **Health and psychosocial instruments (Ovid)**     1. ("social determinant*" or "social need*" or "social risk*" or "determinant* of health" or SDOH).mp. 2. (economic or socioeconomic or poverty or employment or unemployment or financial or "low income").ti,hw. 3. (education or "health literacy").ti,hw. 4. ("social support*" or "support network*" or "social exclusion" or "social network*" or "social isolation" or loneliness or discrimination or racism or incarcerat* or immigra* or refugee*).ti,hw. 5. ((health or healthcare) adj2 access*).ti,hw. 6. (neighborhood or neighbourhood or environment* or housing or transport* or violence).ti,hw. 7. ("food security" or "food insecurity").ti,hw. 8. ("health* behavior*" or "health* behaviour*" or lifestyle or nutrition or malnutrition or diet* or smoking or "physical activity" or exercise or sedentary).ti,hw. 9. (2 and 3 and 4) or (2 and 3 and 5) or (2 and 3 and 6) or (2 and 3 and 7) or (2 and 3 and 8) or (2 and 4 and 5) or (2 and 4 and 6) or (2 and 4 and 7) or (2 and 4 and 8) or (2 and 5 and 6) or (2 and 5 and 7) or (2 and 5 and 8) or (2 and 6 and 7) or (2 and 6 and 8) or (2 and 7 and 8) or (3 and 4 and 5) or (3 and 4 and 6) or (3 and 4 and 7) or (3 and 4 and 8) or (3 and 5 and 6) or (3 and 5 and 7) or (3 and 5 and 8) or (3 and 6 and 7) or (3 and 6 and 8) or (3 and 7 and 8) or (4 and 5 and 6) or (4 and 5 and 7) or (4 and 5 and 8) or (4 and 6 and 7) or (4 and 6 and 8) or (4 and 7 and 8) or (5 and 6 and 7) or (5 and 6 and 8) or (5 and 7 and 8) or (6 and 7 and 8) 10. 1 or 9 |

Grey literature searched 29.10.2024

| SIREN    ‘social’ in search text box  Refine by ‘resource type: tools& toolkits’ |
| --- |
| TRIP    "screening tool*" AND ("social determinant*" OR "social need*" OR "social risk*" OR sdoh)    **Plus**    ("screen*" OR "tool" OR " checklist" OR "needs assessment" OR "questionnaire") AND ("social determinant*" OR "social need*" OR "social risk*" OR "determinant* of health" OR sdoh)  *Order by relevance* |
