# Supplementary material for: Social Needs Screening Tools for Clinical Populations in Australia and New Zealand: A Scoping Review and Critical Analysis
Source: Health Expect. 2026 Feb 26;29(2):e70626. doi: 10.1111/hex.70626 (PMC12936985; doi:10.1111/hex.70626)
Supplement: Supplementary file 2 — Appendix_B. [file HEX-29-e70626-s002.docx]

Appendix B. References of relevant reviews

1. Berkowitz (2023). Health Care's New Emphasis on Social Determinants of Health.
2. Bleacher (2019) The Feasibility of Screening for Social Determinants of Health: Seven Lessons Learned.
3. Cammy H, Boch SJ, Ford J, Liu C, Krist AH (2024). An Examination of Stakeholder-Driven Health-Related Social Needs Screening Tools in Medical Care Delivery.
4. Holt JM, Cushing CC, Packnett ER, Coley KC, Thompson DA, Tobin JN, et al. (2023). Comparison of SIREN social needs screening tools and Simplified Omaha System Terms: informing an informatics approach to social determinants of health assessments.
5. Md (2018). Supporting our patients' health outside of the office.
6. SIREN. Social determinants of health: Guide to social needs screening.
7. SIREN. Screening for social needs: Guiding care teams to engage patients.
8. SIREN. Evaluating social determinants of health: Best practices, opportunities, and resources for collaborative quality initiatives.
9. Andermann A. (2018) Screening for social determinants of health in clinical care: moving from the margins to the mainstream. Public Health Rev.
10. Anonymous. (2018) Towards a global monitoring system for implementing the Rio Political Declaration on Social Determinants of Health: developing a core set of indicators for government action on the social determinants of health to improve health equity. Int J Equity Health. 2018;17(1):136.
11. Boch SJ, Krauter K, Adler N, Browne T. An Integrative Review of Social Determinants of Health Screenings used in Primary Care Settings. Int J Nurs Stud. 2020;110:103689.
12. Brandt CA, Tamura MK, Covinsky KE, Sarkisian CA. Assessing and Addressing Social Determinants of Cardiovascular Health. J Am Heart Assoc. 2023;12(5):e026762.
13. Brown AF, Buitrago D, Raskind IG, Duru OK, Karter AJ, Rodriguez HP, et al. Patient and Patient Caregiver Perspectives on Social Screening: A Review of the Literature. J Gen Intern Med. 2023;38(3):731-42.
14. Davis JL, Buitrago D, Raskind IG, Duru OK, Karter AJ, Rodriguez HP, et al. Collection and Use of Social Determinants of Health Data in Inpatient General Internal Medicine Wards: A Scoping Review. J Gen Intern Med. 2023;38(3):743-54.
15. Dobson R, Giovannoni G. Social determinants of health in multiple sclerosis. Mult Scler Relat Disord. 2022;58:103522.
16. Karran EL, Buitrago D, Raskind IG, Duru OK, Karter AJ, Rodriguez HP, et al. The 'what' and 'how' of screening for social needs in healthcare settings: a scoping review. BMC Health Serv Res. 2023;23(1):193.
17. Kim D, Buitrago D, Raskind IG, Duru OK, Karter AJ, Rodriguez HP, et al. Screening for social determinants of health among populations at risk for MASLD: a scoping review. BMC Public Health. 2024;24(1):1-15.
18. Krzyzanowski MC, Buitrago D, Raskind IG, Duru OK, Karter AJ, Rodriguez HP, et al. The PhenX Toolkit: Measurement protocols for assessment of social determinants of health. Curr Protoc Hum Genet. 2021;108(1):e110.
19. Krzyzanowski MC, Buitrago D, Raskind IG, Duru OK, Karter AJ, Rodriguez HP, et al. The PhenX Toolkit: Measurement Protocols for Assessment of Social Determinants of Health. Curr Protoc Hum Genet. 2023;109(1):e144.
20. LaForge K, Gold R, Cottrell E, Bunce AE, Proser M, Hollombe C, et al. How 6 Organizations Developed Tools and Processes for Social Determinants of Health Screening in Primary Care: An Overview. J Ambul Care Manage. 2018;41(1):2-14.
21. Li J, Buitrago D, Raskind IG, Duru OK, Karter AJ, Rodriguez HP, et al. Realizing the Potential of Social Determinants Data: A Scoping Review of Approaches for Screening, Linkage, Extraction, Analysis and Interventions. J Gen Intern Med. 2024;39(1):1-13.
22. Montayre J, Buitrago D, Raskind IG, Duru OK, Karter AJ, Rodriguez HP, et al. Measuring Social Frailty: A Scoping Review of Available Scales and Tools. J Gerontol A Biol Sci Med Sci. 2024;79(1):1-11.
23. Razon A, Buitrago D, Raskind IG, Duru OK, Karter AJ, Rodriguez HP, et al. Content Analysis of Transportation Screening Questions in Social Risk Assessment Tools: Are We Capturing Transportation Insecurity? J Racial Ethn Health Disparities. 2022;9(5):1481-92.
24. Reyes AM, Buitrago D, Raskind IG, Duru OK, Karter AJ, Rodriguez HP, et al. Interventions Addressing Social Needs in Perinatal Care: A Systematic Review. Matern Child Health J. 2021;25(12):1891-906.
25. Ruiz-Escobar E, Buitrago D, Raskind IG, Duru OK, Karter AJ, Rodriguez HP, et al. Screening and Referral Care Delivery Services and Unmet Health-Related Social Needs: A Systematic Review. J Gen Intern Med. 2021;36(12):3813-25.
26. Stanhope KK, Buitrago D, Raskind IG, Duru OK, Karter AJ, Rodriguez HP, et al. The impact of screening for social risks on OBGYN patients and providers: A systematic review of current evidence and key gaps. Am J Obstet Gynecol. 2023;228(4):439-54.
27. Wong-See D, Buitrago D, Raskind IG, Duru OK, Karter AJ, Rodriguez HP, et al. Clinical Pathways for the Identification and Referral for Social Needs: A Systematic Review. J Gen Intern Med. 2023;38(3):755-67.
28. SIREN. Social determinants of health: Screening in the clinical setting.
29. SIREN. Social determinants of health: Improve health outcomes beyond the clinic walls.
30. SIREN. Social Needs Screening Tool Comparison Table.
31. SIREN. Identifying and addressing social needs in primary care settings.
32. Henrikson NB, Buitrago D, Raskind IG, Duru OK, Karter AJ, Rodriguez HP, et al. Psychometric and Pragmatic Properties of Social Risk Screening Tools: A Systematic Review. J Gen Intern Med. 2023;38(3):768-80.
33. Neshan S, Buitrago D, Raskind IG, Duru OK (2024). Screening tools to address social determinants of health in the United States: A systematic review
34. Suzuki et al. Scoping Review of Screening and Assessment Tools for Social Determinants of Health in the Field of Cardiovascular Disease
