# Supplementary material for: Social Needs Screening Tools for Clinical Populations in Australia and New Zealand: A Scoping Review and Critical Analysis
Source: Health Expect. 2026 Feb 26;29(2):e70626. doi: 10.1111/hex.70626 (PMC12936985; doi:10.1111/hex.70626)
Supplement: Supplementary file 3 — Appendix_C. [file HEX-29-e70626-s003.docx]

Appendix C. Data items and definitions

|  | **Data point** | **Definitions and format** |
| --- | --- | --- |
| **Characteristics** | Covidence ID | Article number assigned in covidence |
|  | First author | First author surname |
|  | Year of publication | Year of publication |
|  | Study title | Title of article |
|  | Origin/country of origin (if applicable) | Country of affiliated organisation, working group or authors e.g., Australia, New Zealand |
|  | Aims/purpose | Copy aims/objectives |
|  | Design | Study design |
|  | Methods | Copy methods summary |
|  | Key findings | Copy key findings |
|  | Name of tool | Name of tool |
|  | Year created | Year tool was created (might be same as year of publication above) |
|  | Development: How was tool developed | Outline development process |
|  | Development: Stakeholder involvement | Outline stakeholder involvement (if any) |
|  | Development: Formal framework | List which SDH framework(s) was used (if any) |
|  | Pilot: Y/N | Has the tool been piloted? |
|  | Pilot: Sample population | Describe sample population demographics |
|  | Pilot: Sample size (n) | Number of participants in pilot study |
|  | Pilot: Setting | Where did the pilot study take place? |
|  | Pilot: Who/how delivered | Who delivered the questionnaire/how was it delivered? |
|  | Evaluation: Y/N | Has the tool been evaluated (i.e. reliability or validity tests)? |
|  | Evaluation: Methods used to validate tool | How the tool was evaluated |
|  | Evaluation: Findings | Brief description of the findings of the evaluation tests |
|  | Number of items in tool (n) | Total number of questions in the tool |
|  | Response options | The type of response options (e.g. tick box, free text) |
|  | Time to administer (minutes) | The time taken to complete the screening tool |
|  | Target population | The population the tool was designed for |
|  | Intended setting | What setting was the tool designed to be administered in |
|  | Target user (doctor, self-administered) | Who the tool is designed to be administered by |
|  | Delivery format (paper, electronic, verbal) | How the tool is designed to be administered |
|  | Proposed intervention(s) | List the proposed intervention corresponding to the social need assessed |
|  | Affiliated organisation | The affiliated organisation or organisation that released or commissioned the guideline. Endorsements do not qualify. |
|  | Funders | List any funding bodies of the document |
|  | Comments | Any comments, notes, queries related to study characteristics. Nil = no comments. |
| **Comprehensiveness** | Covidence ID | Covidence ID |
|  | Name of tool | Name of tool |
|  | Item number | Question number |
|  | Item (verbatim) | Question (start new line per question) |
|  | Response options (verbatim) | Reponse options corresponding to that question. |
|  | Economic stability | If the question assesses any of the subdomains, mark the subdomain(s) with 'X'. Otherwise leave blank. |
|  | Education | If the question assesses any of the subdomains, mark the subdomain(s) with 'X'. Otherwise leave blank. |
|  | Social and community context | If the question assesses any of the subdomains, mark the subdomain(s) with 'X'. Otherwise leave blank. |
|  | Healthcare access | If the question assesses any of the subdomains, mark the subdomain(s) with 'X'. Otherwise leave blank. |
|  | Neighbourhood and physical environment | If the question assesses any of the subdomains, mark the subdomain(s) with 'X'. Otherwise leave blank. |
|  | Food | If the question assesses any of the subdomains, mark the subdomain(s) with 'X'. Otherwise leave blank. |
|  | Health behaviours | If the question assesses any of the subdomains, mark the subdomain(s) with 'X'. Otherwise leave blank. |
|  | Other unspecific domain | If the question assesses any of the subdomains, mark the subdomain(s) with 'X'. Otherwise leave blank. |
|  | Not SDH | If the question assesses any of the subdomains, mark the subdomain(s) with 'X'. Otherwise leave blank. |
| **Actionability** | Covidence ID | Covidence ID |
|  | Name of tool | Name of tool |
|  | Action (whom): State action | State verbatim the behaviour that needs to change (e.g. providing the screening tool to patients for self-completion, or verbally working through the screening tool with patients). Not stated = action not stated. |
|  | Action (whom): Score action (0-2) | 0 = element not mentioned; 1 = element mentioned but nonspecific; 2 = element mentioned and specific detail provided (replicable) |
|  | Actor (who): State actor | State verbatim the person/people that do(es) or could do the action (e.g. administrative staff, or nurse). Not stated = actor not stated. |
|  | Actor (who): Score actor (0-2) | 0 = element not mentioned; 1 = element mentioned but nonspecific; 2 = element mentioned and specific detail provided (replicable) |
|  | Context (where or in what circumstance): State context | State verbatim where or in what circumstance the action should or should not be performed (e.g. in the waiting room, or at intake). Not stated = context not stated. |
|  | Context (where or in what circumstance): Score context (0-2) | 0 = element not mentioned; 1 = element mentioned but nonspecific; 2 = element mentioned and specific detail provided (replicable) |
|  | Target (whom): State target | State verbatim the person/people with/for whom the action is performed (e.g. all patients new to the service). Not stated = target not stated. |
|  | Target (whom): Score target (0-2) | 0 = element not mentioned; 1 = element mentioned but nonspecific; 2 = element mentioned and specific detail provided (replicable) |
|  | Time (when): State time | State verbatim when the action should be performed (time, date, frequency) (e.g. before or during first clinical assessment). Not stated = time not stated. |
|  | Time (when): Score time (0-2) | 0 = element not mentioned; 1 = element mentioned but nonspecific; 2 = element mentioned and specific detail provided (replicable) |
|  | Comments | Any comments, notes, queries related to AACTT assessment. Nil = no comments. |
