# Supplementary material for: Social Needs Screening Tools for Clinical Populations in Australia and New Zealand: A Scoping Review and Critical Analysis
Source: Health Expect. 2026 Feb 26;29(2):e70626. doi: 10.1111/hex.70626 (PMC12936985; doi:10.1111/hex.70626)
Supplement: Supplementary file 4 — Appendix_D. [file HEX-29-e70626-s004.docx]

**Appendix D.** Actionability of each screening tool for social needs, as assessed using the Action, Actor, Context, Target, Time (ACCTT) framework

| **Screening tool** | | **Social Health History Screening Tool Research Project Questionnaire (FUST)** | **Nursing Equity Assessment Tool (NEAT)** | **Steps to Better Health Questionnaire (STBH-Q)** | **Social Determinants of Health Screening Tool (SDoHST)** | **Brief risk factor survey (BRFS)** |
| --- | --- | --- | --- | --- | --- | --- |
| **Action (what)** | **State action** | to provide self-complete questionnaire to patients | nurses to use checklist during consultations to prompt assessment for complex care needs of newly diagnosed patients with cancer | end users to provide their clients with self-report questionnaire | NR | to provide self-complete survey to patients in waiting room |
|  | **Score** | 1 | 2 | 1 | 0 | 1 |
| **Actor (who)** | **State actor** | NR | "specialist cancer nurses" | "clinicians and others interested in measuring social determinants of health at an individual level" "potential end users... members of a medical society and... members of a community services network" | "stakeholders ... likely to be using the screening tool as part of their service provision ... included social workers, nurses, physiotherapists, and researchers" | "survey is acceptable and feasible ... for collection by non-clinical staff" "It can be administered by non-clinical professional staff" |
|  | **Score** | 0 | 2 | 1 | 1 | 1 |
| **Context (where or in what circumstance)** | **State context** | "diverse clinical settings" | "oncology settings" "during consultations" | "a range of settings, such as primary health care or via social prescribing programmes" | "hospital services" | "antenatal clinic waiting rooms" "public waiting rooms of the hospital clinics" |
|  | **Score** | 1 | 2 | 1 | 1 | 2 |
| **Target (whom)** | **State target** | "patient" | "newly diagnosed patients with cancer" | "clients of health professionals" | "patients accessing hospital services" "adults with a chronic disease predominantly treated in hospital" | "pregnant women" |
|  | **Score** | 1 | 2 | 0 | 2 | 2 |
| **Time (when)** | **State time** | "before their first clinical consultation" is in relation to pilot study; unclear on intended timing for questionnaire more broadly | "between first consultation and establishment of treatment plan" | NR | NR | NR |
|  | **Score** | 1 | 2 | 0 | 0 | 0 |

NR = not reported
